# Validation of the Dutch version of the primary care resources and support for self-management tool: A tool to assess the quality of self-management support

**DOI:** 10.1371/journal.pone.0229771

**Published:** 2020-03-10

**Authors:** Maarten Voorhaar, Erik WMA Bischoff, Guus Asijee, Jean Muris, Onno CP van Schayck, Annerika Slok, Anja Visser

**Affiliations:** 1 Maastricht University, CAPHRI Care and Public Health Research Institute, Department of Family Medicine, Maastricht, the Netherlands; 2 Radboud University Medical Centre, Department of Primary and Community Care, Nijmegen, the Netherlands; 3 University of Groningen, Theology and Religious Studies,Groningen, the Netherlands; Uppsala University, SWEDEN

## Abstract

**Introduction:**

Enhancing the self-management activities of patients improves the quality of care and is an integrated element of current healthcare provision. However, self-management support (SMS) is not yet common in healthcare. The Primary Care Resources and Support for Self-Management (PCRS) is a tool for healthcare professionals to assess the quality of SMS. In this study, we assessed the validity and reliability of the Dutch version of the PCRS.

**Method:**

The validation of the PCRS was performed in Dutch healthcare centres. Correlations between the PCRS scores and the Assessment of Chronic Illness Care (ACIC) and Clinician Support for Patient Activation Measure (CS-PAM) scores were calculated to assess the convergent and discriminant validity. A confirmatory factor analysis (CFA) was performed to test the factor structure. Lastly, the internal consistency and face validity were assessed.

**Results:**

The convergent and discriminant validity were good, with respective correlations of 0.730 (p < 0.001) and 0.030 (p > 0.050) between the PCRS and the ACIC SMS subscale and the PCRS and the CS-PAM. Although 49% of the variance of the PCRS was explained by one factor, the CFA could not confirm a fit between a one-factor model and the data. The reliability was excellent (Cronbach’s α = 0.921).

**Conclusion:**

The PCRS showed good validity and excellent internal consistency. However, the evidence for its validity was inconclusive. We therefore suggest rephrasing specific items.

## Introduction

In chronic care, the ultimate goal is to minimise the experienced burden of disease while maximising the quality of life. One important aspect of reaching this goal, independent of which chronic conditions patients might have, is self-management by patients. Patients who have better self-management skills report better quality of life, consider themselves to be in better control of their disease and have a healthier lifestyle [1]. Furthermore, to relieve the burden on healthcare providers, care is considered to be more efficient when patients are more engaged with their healthcare provider and share responsibilities and decisions [2,3]. As healthcare costs are increasing while the number of healthcare providers per inhabitant is decreasing and the digital possibilities for patients are growing, there is a need for patients to engage in more self-management activities. Self-management in chronic care is defined as a patient’s ability to define and solve problems, set priorities and establish goals by creating treatment plans. Rather than an isolated set of tasks by patients, it is a collaboration between healthcare provider and patient. It is expected that, by encouraging self-management behaviour, active participation will improve, resulting in better perceived health [4,5,6]. In the Netherlands, self-management is considered to be a key recommendation in patient-centred care standards [7] and treatment guidelines in chronic care [8,9].

Many patients lack the ability to understand their illness and treatment or are unable to make optimal decisions relating to their care [10]. To optimise self-management, healthcare professionals, and the healthcare organisations for which they work, need to incorporate self-management support (SMS) into daily care. Optimal SMS is achieved when healthcare providers are skilled in and actively integrate SMS into everyday care [11,12].

From an organisational perspective, evidence shows that programmes based on the Chronic Care Model (CCM) improve the quality of healthcare delivery and result in more affordable healthcare [13–16]. The Chronic Care Model (CCM) identifies the essential elements of a healthcare system that encourage high-quality chronic disease care: the community; the health system; self-management support; delivery system design; decision support; and clinical information systems. The quality of care based on the CCM is specifically measured by the Assessment of Chronic Integrated Care (ACIC) questionnaire. Self-management is a core element of this model, aiming to enhance patient participation.

While the ACIC assesses all the relevant aspect of chronic care provision, the Primary Care Resources and Support for Chronic Disease Self-Management (PCRS) was developed specifically for SMS. The objective of the PCRS is to identify the strengths and gaps in resources, services and support within healthcare organisations, resulting in an assessment of the quality of SMS. This assessment enables healthcare organisations to improve the SMS provided by healthcare professionals. After the completion of this instrument, areas for improvement can be identified and prioritised to address these areas [17]. The PCRS has been used by healthcare organisations worldwide, and currently US, UK and Spanish versions are available [18]. However, little evidence on the validity and reliability of this instrument exists [17]. In this study, we assessed the validity and reliability of a Dutch translation of the PCRS as a tool to measure the quality of SMS in the Netherlands. As self-management is relevant not only to primary care but also to secondary and tertiary care, the research topic was extended to speciality care.

## Method

Between November 2013 and March 2014, healthcare professionals were recruited from different Dutch healthcare centres. As no clear guidance on sample size calculations for validation studies of self-reported questionnaires exists [19], it was decided to choose a sample of 60 participants, similar to the number of participants involved in the reliability assessment of the PCRS [17]. The participants provided written informed consent and were asked to complete the PCRS, the Assessment of Chronic Integrated Care (ACIC) and the Clinician Support for Patient Activation Measure (CS-PAM). In addition, questions regarding age, job function, number of years active in healthcare and type of organisation were posed. Two weeks after the completion of the initial questionnaire, all the participants were asked to complete the PCRS for a second time.

This study did not require approval from a medical–ethical committee as no patients were involved and no intervention was under investigation. It was registered in the Dutch clinical trial registry (www.trialregister.nl) under number NTR4419.

### Questionnaires

#### Primary Care Resources and Support for Self-Management (PCRS)

The PCRS is a self-reported questionnaire completed by healthcare professionals. The aim of the PCRS is to measure and improve the quality of SMS of patients with a chronic condition [17,20]; see S1 Fig. for details. The PCRS consists of two subscales–Patient Support (PS) and Organisational Support (OS)–containing eight items each. Each item is rated on a ten-point scale within four quality levels. The four levels (D, C, B and A, respectively low to high) indicate the consistency, level of implementation and integration of SMS into everyday chronic care. Except for level D, scores on a specific level indicate a certain degree of quality of SMS (three possible scores per level). Scores on level D mean that this specific topic is not part of everyday care at all, and only one score is possible. The original PCRS was developed and evaluated through an iterative process, and the psychometric properties are limited to the reliability scores of the PS and OS subscales only. The Cronbach’s α values for the PS and OS subscales are .94 and .90, respectively, which are considered to be highly reliable [17].

For this study, the UK version of the PCRS was translated into Dutch. The translation process was performed in concordance with the World Health Organization’s (WHO) recommendations for the translations of questionnaires [21]. First, the English version was translated into Dutch by a certified translator. Second, this first draft version was evaluated by a team of selected healthcare providers from primary, secondary and tertiary care organisations as well as by a patient representative. They assessed the content of the questionnaire for understandability and relevance to Dutch healthcare. Based on their recommendations, the questionnaire was adjusted. Third, the adjusted version was translated back into English by a second qualified translator who was not involved in the initial translation. Fourth, to assess possible major relevant differences between the two versions, items from the original PCRS UK were compared with the back-translated version by a third certified translator and, independently, by one of the authors.

#### Assessment of Chronic Integrated Care (ACIC)

The ACIC is a self-reported questionnaire completed by healthcare professionals and used to assess the quality of the organisation of chronic care based on the Chronic Care Model (CCM) [22]; see S2 Fig. for details. As chronic care in the Netherlands is based on the CCM, the ACIC is considered to be appropriate to assess the quality of healthcare. The questionnaire consists of thirty-four items divided into six subscales, representing the six key components of the CCM (healthcare systems, community, self-management support, decision support, clinical information systems and delivery design). Similar to the PCRS, items are scored within four levels (D to A), and the degree within each level is scored by ticking one of three items. Different from the PCRS is the possibility to tick one of three scores on the lowest level (D), resulting in twelve possible scores (one to twelve) per item. Higher scores indicate better implementation of the CCM in everyday care. We used the Dutch version of the ACIC, which has satisfactory psychometric properties comparable to the original ACIC; the reliability of the subscales as measured by Cronbach’s α varied between 0.70 and 0.86 [23].

For validation purposes, the Self-Management Support subscale (SMS) of the ACIC was used in this study. The ACIC SMS aims to assess the quality of SMS but is less comprehensive than the PCRS [17]. Furthermore, the ACIC SMS was designed to assess the quality of SMS provided on the patient level but not on the organisational level. The PCRS is therefore considered to complement the ACIC SMS by providing more detailed information.

#### Clinician Support for Patient Activation Measure (CS-PAM)

The CS-PAM is a commercially available self-reported questionnaire completed by healthcare professionals to measure their perception of patient activation [24]; see S3 Fig. for details. It measures how valuable patient participation is according to the healthcare professionals. Patient activation is considered to be an indicator of self-management, and higher scores on the CS-PAM indicate that patients are believed to be able to self-manage their condition better. The CS-PAM consists of 14 items scored on a 5-point Likert scale; see S3. The Dutch version has overall acceptable psychometric properties; the reliability as measured by Cronbach’s α varies between 0.81 and 0.97 [25]. While the CS-PAM assesses clinicians’ beliefs and attitudes about the importance of patient self-management, the PCRS measures the quality of self-management support. We consider these measures to be different in their scope; the first relates to the opinion of the healthcare provider on a specific patient or a patient population, while the latter is a judgement of the organisation of self-management support.

#### Validity measures

The construct validity was assessed by determining both convergent and discriminant validity. Additionally, the factor structure of the PCRS was tested. The convergent validity was assessed by calculating Spearman’s rank order correlation between the PCRS total score and the total score on the ACIC SMS subscale. A correlation of > 0.70 is considered to be acceptable evidence of convergent validity.

The discriminant validity was assessed by calculating Spearman’s rank order correlation between the PCRS total score and the CS-PAM score, in which the factual SMS was expected to differ from the healthcare professional’s beliefs on the importance of patients’ self-management behaviour and competencies. A correlation of < 0.35 is considered to be acceptable evidence of discriminant validity.

Confirmatory factor analysis (CFA) was performed to assess how well the measured variables represent the expected constructs [26] Two models were considered: a one-factor model for the PCRS, as it was expected that the PCRS measures one construct, namely SMS, and a two-factor model for the PCRS subscales, as it was expected that the PCRS consists of two independent but related constructs, specifically PS and OS. Three goodness-of-fit indices were calculated to assess the global fit of the model, that is, the chi-square statistic, the comparative fit index (CFI) and the root mean square error of approximation (RMSEA). In the chi-square test, values closer to 0 indicate a better fit. A CFI value > 0.90 and an RMSEA value < 0.05 indicate a psychometrically acceptable fit of the data [27].

The face validity was assessed through a rating procedure in which the participants were requested to rate the understandability and relevance of each item of the PCRS. The rating for understandability was as follows: 1 = it is not clearly understood what is meant by this item, 6 = it is somewhat understood what is meant by this item and 10 = it is completely understood what is meant by this item. The rating for relevance was as follows: 1 = completely irrelevant, 6 = somewhat relevant and 10 = completely relevant. Mean (SD) scores were calculated per item.

The internal consistency was assessed by measuring composite reliability, using CFA to calculate factor loadings for individual items. Factor loadings are considered to be internally consistent with a value > 0.7 [28]. Furthermore, Cronbach’s α was calculated. Generally, if Cronbach’s α > 0.9, the internal consistency is considered to be excellent. The internal consistency is considered to be good if Cronbach’s α is between 0.70 and 0.90. Finally, test–retest reliability was assessed by comparing the results of the first scores of the PCRS with the scores of the PCRS that was completed two weeks later.

Statistical analyses were performed using the Statistical Package for the Social Sciences V.20.0 (SPSS, Chicago, Illinois, USA). The results were considered to be statistically significant at the P < 0.05 level.

## Results

### Participants

Out of the 63 participants invited, 47 completed the first questionnaire (t = 0), of whom 40 also completed the PCRS at t = 1. The data of 1 participant were inadequate for further analysis, as 1 questionnaire was not completed. Table 1 shows the characteristics of the 46 participants.

**Table 1:** Overview of participant characteristics.

	N (%)
**Function **	
**(Family) physician**	8 (17)
**Nurse**	13 (28)
**Paramedic**	16 (35)
**Psychosocial care professional**	7 (15)
**Pharmacist**	1 (2)
**Quality assurance specialist**	1 (2)
**Setting **	
**General practice**	15 (33)
**Hospital**	6 (13)
**Specialised diagnostic/care centre**	25 (54)
**Age **	
**18–30**	10 (22)
**31–50**	24 (52)
**> 51**	11 (24)
**Undefined**	1 (2)
**Years of working experience **	
**0–10**	15 (33)
**11–20**	13 (28)
**> 21**	18 (39)

### Convergent and discriminant validity

The PCRS total score correlated strongly and significantly with the total ACIC SMS score (r = 0.730, p < 0.001). In addition, the scores of the PS and OS subscales correlated strongly and significantly with the ACIC SMS score (r = 0.722, p < 0.001 and r = 0.552, p < 0.001, respectively). The correlations between the PCRS total score and the PCRS PS and PCRS OS subscales with all the other ACIC subscales were less strong than the correlations with the ACIC SMS subscale, yet they were still significant. The strongest correlation between the PCRS total score subscales besides the ACIC SMS subscale was with the ACIC clinical information systems and the ACIC integration of CCM subscales (r = 0.589, p < 0.05 and r = 0.617, p < 0.05, respectively). The correlations between all the individual items of the PCRS PS subscale with the ACIC SMS subscale were significant and stronger than those with the other ACIC subscales (r = 0.444 to r = 0.695). All the items from the PCRS OS subscale correlated significantly with the ACIC SMS subscale as well (r = 0.295 to r = 0.569). However, four of the items (items 9, 14, 15 and 16) correlated more strongly with other ACIC subscales (healthcare organisation, clinical information system and integration of CCM components).

The correlation between the PCRS total score and the CS-PAM score was low and not significant (r = 0.225, p = 0.147). The correlations between the PS and OS subscales and the CS-PAM score were also low and not significant (r = 0.264, p = 0.087 and r = -0.196, p = 0.209, respectively).

### Factor analysis

Confirmatory factor analysis (CFA) was performed to investigate the factor structure of the PCRS. Neither the one-factor model (chi-square (df = 104) = 171.865, p < 0.001) nor the two-factor model (chi-square (df = 103) = 170.435, p < 0.001) fitted the data. This was confirmed by the other indices, as, for both models, the CFI did not exceed 0.90 and the RMSEA > 0.06 (Table 2). However, principal component analysis suggested a one-factor solution, with this factor explaining 49% of the variance (see Table 3), while the other two found components with eigenvalues > 1 that explained 16%.

**Table 2:** Model fit indexes of the structural equation modelling.

	Chi-square	CFI	RMSEA
Model 1: one-factor model	171.865*	.840	.120
Model 2: two-factor model	170.435*	.841	.121

**Table 3:** Model fit indexes of the structural equation modelling.

Component	Eigenvalue	Percentage of variance
1	7.845	49.034
2	1.397	8.732
3	1.192	7.452

In model 1, the factor loadings for nine of the sixteen items were below the cut-off score of 0.70. This was the case for all eight items from the OS subscale. In model 2, the individual factor loadings showed larger average loadings for the PS subscale than for the OS subscale. The average factor loadings for all the items of the OS subscale as well as the factor loadings for six of the eight items of this subscale were below the cut-off score of 0.70. Similar to model 1, items 11, 12 and 16 showed poor internal consistency with the OS subscale, while items 10 and 13 correlated with the PS subscale. Items 11, 12 and 16 refer to the extent to which ongoing quality improvement is present, a system for the documentation of SMS services is in place and doctors and team and staff members receive education and training on self-management, respectively. Item 10 refers to the extent to which referrals are coordinated between primary care and specialist, whereas item 13 concerns the extent to which patient input is integrated into care provision.

### Face validity

Most items were rated as ‘completely understandable and relevant’ to the assessment of SMS. Two items, 12 and 14, were rated as ‘not clearly understandable’ by 20% and 13% of the participants, respectively. Furthermore, items 12 and 14 were rated as ‘irrelevant’ by 17% and 10% of the participants, respectively. Item 12 is a question on the documentation of self-management support, while item 14 is a question regarding the extent to which SMS is integrated into daily care. All the other items were rated as ‘somewhat’ or ‘completely understandable and relevant’ by more than 90% of the participants.

### Internal consistency

Internal consistency, as indicated by Cronbach’s α, was high and reached the required threshold (r > 0.90). Regarding the test–retest reliability, the correlations between the total PCRS score and the scores of the two PS and OS subscales at t = 0 and t = 1 were strong and significant (Table 4). The average total scores at t = 1 were slightly higher than those at t = 0.

**Table 4:** Internal consistency and correlation between total and domain PCRS scores for test–retest reliability.

	Cronbach’s α	PCRS Patient Support t = 1	PCRS Organisational Support t = 1	PCRS total t = 1^a^
PCRS Patient Support t = 0	.931*	.881*		
PCRS Organisational Support t = 0	.763*		.653*	
PCRS total t = 0	.921*			.774*

^a^ Spearman rank-order correlation

* p < .001

## Discussion

We found that the Dutch PCRS showed good convergent, discriminant and face validity and excellent test–retest reliability. The PCRS correlated strongly and significantly with the ACIC-SMS subscale but only weakly and non-significantly with the CS-PAM. Most of the individual items were considered to be understandable and relevant to assessing self-management support. The test–retest and reliability measures were high and might suggest that the PCRS measures one factor, SMS. However, the CFA showed that neither a one-factor nor a two-factor model fitted the data. Items from the Organisational Support (OS) subscale appeared to be internally inconsistent with the construct that it was supposed to measure (SMS) as well as with the total scale. There also seemed to be a correlation between two items from the OS scale with the Patient Support (PS) scale, indicating a possible overlap between the two subscales.

Inconsistency was found in the results for internal consistency. As Cronbach’s α is often used as a measure for internal consistency, the results for internal consistency contradict the CFA and the factor loadings found. However, there is evidence to limit the interpretation of a high α. Sijtsma [29] reported that Cronbach’s α is rather a lower-bound estimate of reliability at best and should not be considered as a measure of internal consistency. Based on this view, Cronbach’s α can be high even when items measure unrelated latent constructs. As the CFA could not fit a one-factor model with the data, the number of latent factors is unknown. Therefore, the appropriateness of Cronbach’s α as an estimate for internal consistency is doubtful. Furthermore, we found that the PCRS and its subscales are significantly related not only to SMS but also to the other six components of the ACIC. This might explain why a fit with one or two factors was not found, as the six components cover related yet distinct aspects of chronic care organization. However, the OS subscale was intended to assess the organisational support for self-management. It can be argued that a single construct (SMS) for construct validity is insufficient. As can be concluded from the results, there are other constructs from the CCM related to the organisation of SMS. Another explanation might be that, for the OS subscale, there was more variability in the items’ understandability and their relevance to measuring SMS. In contrast, items from the PS subscale were well understood, relevant and internally consistent and do not need to be revised. Although the items from the OS subscale aim to assess SMS, the wording of most items is not specific to self-management. Therefore, these items could be related to other aspects of chronic care, which might lead to different interpretations of the content of these items. An adjustment of items 12 and 14, followed by revalidation of the understandability and relevance of these items, is advised before using the PCRS in daily practice. Even though the translation process was performed according to guidelines for translating questionnaires, we cannot exclude the possibility that the translations of items 12 and 14 were interpreted differently from expected by the participants. As we cannot rule this out, the fact that we were unable to find one or two factors with the CFA might be explained by this finding, and the interpretation of the CFA is therefore ambiguous.

A strong point of this study is that, to our knowledge, this is the first attempt to provide evidence for the validity and reliability of the PCRS. Even though the PCRS is available for the UK, the USA and Spain, no literature on its validity and reliability has been published. The results from our study support the validity and reliability of the PCRS. However, there seems to be a discrepancy between the results of the measures used.

The representation of different healthcare providers is both a strong point and a weakness of this study. As patients with a chronic condition are increasingly managed by multidisciplinary teams of healthcare providers, such as GPs, GP nurses, physiotherapists and psychologists, the views on healthcare provision of all healthcare professionals are equally important. A downside of this approach is, consequently, the small sample size of this study. This limits the interpretation of the data, as the subgroups based on medical speciality are too small for statistical sub-group analysis, which will not allow meaningful conclusions. The lack of model fit may be explained by this small sample size. However, the sample size itself as an explanation for the poor model fit is ambiguous. By using both a larger total sample size and a larger sample size per speciality and healthcare setting, it should be possible to rule out the impact of a small sample size on the results of the factor analysis.

Furthermore, it is recommended to study the relationship between SMS as measured by the PCRS and SMS perceived by patients, for instance measured using the Patient Activation Measure [30]. A positive correlation between SMS provided by healthcare professionals and SMS perceived by patients would imply the possibility of a causal relationship, whereby better SMS will lead to better self-management by patients.

An omission in this study was the registration of the sex of the participants. This prevented us from determining whether male and female healthcare providers had different insights into SMS.

The results from this study may suggest that the PCRS can appropriately measure SMS, but the evidence is not conclusive. Before this tool can be used in daily practice or research, further improvement of the OS subscale, by rewriting at least items 12 and 14, is recommended. Lastly, we recommend studying the effect of the use of the PCRS on improvements in healthcare provision, experienced health and healthcare use. We therefore suggest rewriting the two items in collaboration with healthcare providers and patients and incorporating these into the PCRS. In future studies on self-management support and organisation of care, we will use the PCRS for specific chronic conditions, such as COPD, to find further and more robust evidence on the validity of the Dutch PCRS.

## Conclusions

The Dutch PCRS showed good convergent, discriminant and face validity and excellent test–retest reliability. However, the evidence for its construct validity, as determined through CFA, was less pronounced, leading to an inconclusive assessment of the validity of the PCRS. We propose that items from the OS subscale should be rephrased and reassessed in a larger sample within distinct therapeutic areas in future studies.

## Supporting information

S1 Fig (DOCX)

S2 Fig (DOCX)

S3 Fig (DOCX)

S1 File PCRS data overview final PO.xlsx. (XLSX)
