# Supplementary material for: Validation of the Dutch version of the primary care resources and support for self-management tool: A tool to assess the quality of self-management support
Source: PLoS One. 2020 Mar 10;15(3):e0229771. doi: 10.1371/journal.pone.0229771 (PMC7064186; doi:10.1371/journal.pone.0229771)
Supplement: S1 Fig — (DOCX) [file pone.0229771.s001.docx]

Supplement 1 (S1): PCRS questionnaire

| Characteristic | | D | C | | | B | | | | | | A | | | | |
| --- | --- | --- | --- | --- | --- | --- | --- | --- | --- | --- | --- | --- | --- | --- | --- | --- |
| 1 | Individualized Assessment of Patient’s Self- Management Educational Needs | …is not done | …is not standardized and/ or does not consistently include most self-management components | | | …is standardized, fairly comprehensive and documented prior to initial goal setting; takes into account language, literacy and culture; assesses patient’s self- management knowledge, behaviors, confidence, barriers, resources, and learning preferences | | | | | | …is an integral part of planned care for chronic disease patients; results are documented, systematically reassessed and utilized for planning with patients | | | | |
|  |  | 1 | 2 | 3 | 4 | 5 | | 6 | | 7 | | 8 | | 9 | | 10 |
| 2 | Patient Self-Management Education | …does not occur | …occurs sporadically or without tailoring to patient skills, culture, educational needs, learning styles or resources | | | …plan is developed with patient (and family if appropriate) based on individualized assessment; is documented in the patient’s notes; all team members generally reinforce same key messages | | | | | | …is documented in patients’ notes; is an integral part of the care plan for patients with chronic diseases; involves family and community resources; is systematically evaluated for effectiveness | | | | |
|  |  | 1 | 2 | 3 | 4 | 5 | | 6 | | | 7 | 8 | 9 | | 10 | |
| 3 | Goal Setting/ Action Planning | …is not done | …occurs but goals are established primarily by health care team rather than developed collaboratively with patients | | | …is done collaboratively with all patients/ families and member(s) of their health care team; goals are specific, documented and available to any team member; goals are reviewed and modified periodically | | | | | | …is an integral part of care for patients with chronic diseases; goals are systematically reassessed and discussed with patients; progress is documented in patients’ notes | | | | |
|  |  | 1 | 2 | 3 | 4 | 5 | 6 | | 7 | | | 8 | 9 | | 10 | |
| 4 | Problem-Solving Skills | …are not taught or practiced with patients | …are taught and practiced sporadically or used by only a few team members | | | … are routinely taught and practiced using evidence-based approaches and reinforced by members of the health care team | | | | | | …. is an integral part of care for people with chronic diseases; takes into account family, community and environmental factors; results are documented and routinely used for planning with patients | | | | |
|  |  | 1 | 2 | 3 | 4 | 5 | 6 | | 7 | | | 8 | 9 | | 10 | |
| 5 | Emotional Health | …is not assessed | …is not routinely assessed; screening and treatment protocols are not standardized or are nonexistent | | | …assessment is integrated into practice and pathways established for treatment and referral; patients are actively involved in goal setting and treatment choices; team members reinforce consistent goals | | | | | | …systems are in place to assess, intervene, follow up and monitor patients’ progress and coordinate among providers; standardized screening and treatment protocols are used | | | | |
|  |  | 1 | 2 | 3 | 4 | 5 | 6 | | 7 | | | 8 | 9 | | 10 | |
| 6 | Patient Involvement | …does not occur | …is passive; clinician or educator directs care with occasional patient input | | | …is central to decisions about self- management goals and treatment options; is encouraged by health care team and office staff | | | | | | … is an integral part of the system of care; is explicit to patients; is accomplished through collaboration among patients and team members; takes into account environmental, family, work or community barriers and resources | | | | |
|  |  | 1 | 2 | 3 | 4 | 5 | 6 | | 7 | | | 8 | 9 | | 10 | |
| 7 | Patient Social Support | …is not addressed | …is discussed in general terms, not based on an assessment of patient’s individual needs or resources | | | …is encouraged through collaborative exploration of resources available to meet individual needs (e.g., significant others, education groups, support groups) | | | | | | … systems are in place to assess needs, link patients with services and follow up on social support plans using household, community, or other resources | | | | |
|  |  | 1 | 2 | 3 | 4 | 5 | 6 | | 7 | | | 8 | 9 | | 10 | |
| 8 | Linking to Community Resources | …does not occur 1 | …is limited to a list or pamphlet of contact information for relevant resources | | | …occurs through a referral system; team discusses patient needs, barriers and resources before making referral | | | | | | …systems are in place for coordinated referrals, referral follow-up and communication among practices, resource organizations and patients | | | | |
|  |  | 1 | 2 | 3 | 4 | 5 | 6 | | 7 | | | 8 | 9 | | 10 | |
| 9 | Continuity of Care | …does not exist | …is limited; some patients have an assigned primary care provider (PCP); planned visits and routine lab work occur sporadically | | | …is achieved through assignment of patients to a PCP or designated primary care team member, scheduling of routine planned visits with appropriate team members, and involvement of most team members in ensuring patients meet care guidelines | | | | | | …systems are in place to support continuity of care, to assure all patients are assigned to a provider or team member, to schedule planned visits and to track and follow up on all patient visits and labs | | | | |
|  |  | 1 | 2 | 3 | 4 | 5 | 6 | | 7 | | | 8 | 9 | | 10 | |
| 10 | Coordination of Referrals | ...does not exist | ... is sporadic, lacking systematic follow-up, review or incorporation into the patient’s care plan | | | …occurs through team and office staff working together to document, track and review completed referrals and coordinate with specialists in adjusting the patient’s care plan | | | | | | …is accomplished by having systems in place to track incomplete referrals and follow up with patients and/ or specialists to complete referrals | | | | |
|  |  | 1 | 2 | 3 | 4 | 5 | 6 | | 7 | | | 8 | 9 | | 10 | |
| 11 | Ongoing Quality Improvement (QI) | … does not exist | ...is possible because organized data are available, but practice has not initiated specific QI projects in this area | | | …is accomplished by a patient care team that uses data to identify trends and launches QI projects to achieve measurable goals | | | | | | … uses a registry, electronic medical record or other system to routinely track key indicators of measurable outcomes; is done through a structured and standardized process with administrative support and accountability to management | | | | |
|  |  | 1 | 2 | 3 | 4 | 5 | 6 | | 7 | | | 8 | 9 | | 10 | |
| 12 | System for Documentation of Self-Management Support Services | …does not exist | …is incomplete or does not promote documentation (e.g., no forms in place) | | | …includes charting or documentation of care plan and self- management goals; is used by the team to guide patient care | | | | | | ... is an integral part of patient medical records; information is easily accessible to all team members and organized to see progression; charting or documentation includes care provided by all care team members and referral specialists | | | | |
|  |  | 1 | 2 | 3 | 4 | 5 | 6 | | 7 | | | 8 | 9 | | 10 | |
| 13 | Patient Input | ... does not occur | ... mechanisms exist, but are not promoted; input solicited sporadically | | | .... is solicited through focus groups, surveys, suggestion boxes, or other means for both service and service delivery improvements under consideration; patients are made aware of mechanisms for input and invited or encouraged to participate | | | | | | …is an essential part of management’s decision-making process; systems are in place to ensure consumer input regarding practice policies and service delivery; there is evidence that management acts on the information | | | | |
|  |  | 1 | 2 | 3 | 4 | 5 | 6 | | 7 | | | 8 | 9 | | 10 | |
| 14 | Integration of Self- Management Support into Primary Care | …. does not exist | …is limited to special projects or to select teams | | | …is routine throughout the practice; team members reinforce consistent strategies | | | | | | ...is built into the practice’s strategic plan; is routinely monitored for quality improvement and visibly supported by leadership | | | | |
|  |  | 1 | 2 | 3 | 4 | 5 | 6 | | 7 | | | 8 | 9 | | 10 | |
| 15 | Patient Care Team (internal to the practice) | … does not exist | …exists but little cohesiveness among team members | | | ...is well defined; each member has defined roles and responsibilities; there is good communication and cohesiveness among members; members are cross-trained, have complementary skills | | | | | | ...is a concept embraced, supported and rewarded by the senior leadership; “teamness” is part of the system culture; case conferences and/or multidisciplinary team reviews are regularly scheduled | | | | |
|  |  | 1 | 2 | 3 | 4 | 5 | 6 | | 7 | | | 8 | 9 | | 10 | |
| 16 | Doctor, Team and Staff Self- Management Education & Training | … does not occur | ...occurs on a limited basis without routine follow-up or monitoring | | | …is provided for some team members using established and standardized curricula; practice assesses and monitors performance | | | | | | ...is supported and incentivized for all key team members; continuing education is routinely provided to maintain knowledge and skills; job descriptions reflect skills and orientation to self management | | | | |
|  |  | 1 | 2 | 3 | 4 | 5 | 6 | | 7 | | | 8 | 9 | | 10 | |
