# Supplementary material for: Validation of the Dutch version of the primary care resources and support for self-management tool: A tool to assess the quality of self-management support
Source: PLoS One. 2020 Mar 10;15(3):e0229771. doi: 10.1371/journal.pone.0229771 (PMC7064186; doi:10.1371/journal.pone.0229771)
Supplement: S2 Fig — (DOCX) [file pone.0229771.s002.docx]

**Supplement 2 (S2): ACIC questionnaire**

Users should complete the ACIC for one chronic condition at a time (e.g., how well they are providing care for diabetes). Respondents (practice teams, health plan representatives) are asked to rate the degree to which each component (e.g., partnerships with community organizations, patient treatment plans) is being implemented within their system for that chronic condition, using a scale ranging from 0 (not at all) to 11 (fully).

Part 1: Organization of healthcare delivery system;

1. Overall organizational leadership in chronic illness care

2. Organizational goals for chronic care

3. Improvement strategy for chronic illness care

4. Incentives and regulations for chronic illness care

5. Senior leaders

6. Benefits

Part 2: Community linkage;

7. Linking patients to outside resources

8. Partnership with community organizations

9. Regional health plans

Part 3a: Self-management support

10. Assessment and documentation of self-management needs and activities

11. Self-management support

12. Addressing concerns of patients and families

13. Effective behavior change interventions and peer support

Part 3b: Decision support

14. Evidence-based guidelines

15. Involvement of specialists in improving primary care

16. Providing education for chronic illness care

17. Informing patients about guidelines

Part 3c: Delivery system design

18. Practice team functioning

19. Practice team leadership

20. Appointment system

21. Follow-up

22. Planned visits for chronic illness care

23. Continuity of care

Part 3d: Clinical information system

24. Registry (list of patients with specific conditions)

25. Reminders to providers

26. Feedback

27. Information about relevant subgroups of patients needing services

28. Patient treatment plans

Part 4: Integration of chronic care components

29. Informing patients about guidelines

30. Information systems/ registries

31. Community programs

32. Organizational planning for chronic illness care

33. Routine follow-up for appointments patient assessments and goal planning

34. Guidelines for chronic illness care
