# Supplementary material for: Validation of the Dutch version of the primary care resources and support for self-management tool: A tool to assess the quality of self-management support
Source: PLoS One. 2020 Mar 10;15(3):e0229771. doi: 10.1371/journal.pone.0229771 (PMC7064186; doi:10.1371/journal.pone.0229771)
Supplement: S3 Fig — (DOCX) [file pone.0229771.s003.docx]

**Supplement 3 (S3): CS-PAM questionnaire**

As a clinical, how important is to you that your patients with long term conditions:

1. Are able to take actions that will help prevent or minimize symptoms associated with their health condition

2. Are able to make and maintain lifestyle changes needed to manage their long term condition

3. Understand which of their behaviours make their condition better and which ones make it worse

4. Can follow through on medical treatments you told them they need to do at home

5. Know what each of prescribed medications does

6. Is able to determine when they need to go to a medical professional for care and when they can manage the problem on their own

7. Are able to work out solutions when new situations or problems arise with their health condition

8. Want to be involved as a full partners with you in making decisions about care

9. Tell you concerns they have about their health even when you do not ask

10. Want to know what procedures or treatments they will receive and why before the treatments are performed

11. Understand the different medical treatments options available for their long term condition

12. Look for trustworthy sources of information about their health and health choices such as on the web, news or books

13. Bring a list of questions when they come to the clinic
